# Hemodynamic Consequences of Hypertrophic Cardiomyopathy with Midventricular Obstruction: Apical Aneurysm and Thrombus Formation

**DOI:** 10.4172/2329-9126.1000161

**Published:** 2014-05-26

**Authors:** Shahryar G Saba, Andrew W Ertel, Michael Siegenthaler, Edward Bodurian, Peter Kellman, Marcus Y Chen, Andrew E Arai, W Patricia Bandettini

**Keywords:** Hypertrophic cardiomyopathy, Midventricular hypertrophy, Midcavity obstruction, Aneurysmal apical chamber, Paradoxic diastolic jet flow in hypertrophic cardiomyopathy, Cardiovascular magnetic resonance imaging of hypertrophic cardiomyopathy

## Abstract

**Background:**

Hypertrophic cardiomyopathy (HCM) with midventricular hypertrophy is an uncommon phenotypic variant of the disease. Midventricular hypertrophy predisposes to intracavitary obstruction and downstream hemodynamic sequelae.

**Case report:**

We present a case of HCM with midventricular hypertrophy and obstruction diagnosed after a CT scan of the abdomen incidentally revealed a filling defect in the left ventricular apex. Transthoracic echocardiography demonstrated mid left ventricular hypertrophy and obstruction, as well as an aneurysmal apex containing a large thrombus. Cardiovascular MRI showed a spade-shaped left ventricle with midcavitary obliteration, an infarcted apex and regions of myocardial fibrosis. Due to the risk of embolization and a relative contraindication to anticoagulation, the patient underwent surgery including thrombectomy, septal myectomy and aneurysmal ligation.

**Conclusions:**

Hypertrophic cardiomyopathy with midventricular hypertrophy leads to cavity obstruction, increased apical wall tension, ischemia and ultimately fibrosis. Over time, patchy apical fibrosis can develop into a confluent scar resembling a transmural myocardial infarction in the left anterior descending coronary artery distribution. Aneurysmal remodeling of the left ventricular apex potentiates thrombus formation and risk of cardioembolism. For these reasons, hypertrophic cardiomyopathy with midventricular obstruction portends a particularly poor prognosis and should be recognized early in the disease process.

## Introduction

Hypertrophic cardiomyopathy is defined as a myocardial disease characterized by unexplained left ventricular hypertrophy in association with non-dilated ventricular chambers [1]. The disease affects approximately 1:500 individuals and has a known genetic basis. Multiple morphologic variants exist, ranging from a normal phenotype without increased wall thickness, to asymmetric septal, midventricular, apical and concentric patterns of left ventricular hypertrophy [2]. Hypertrophic cardiomyopathy with mid ventricular obstruction, defined as an intracavitary gradient ≥ 30 mm Hg, affects approximately 10% of patients with the disease, as determined in a large Japanese cohort [3]. Hypertrophic cardiomyopathy with midventricular obstruction and concurrent akinetic or aneurysmal apical chamber affects even fewer patients, ranging from approximately 1–3% of all individuals with HCM [3,4]. Patients with midventricular obstruction are significantly more likely to develop an apical aneurysm compared to those without an intracavitary gradient (28% versus 1.8%, p<0.001) [3]. The presence of an aneurysmal apex in association with midventricular obstruction portends a poor prognosis, and may be an underappreciated variant of the disease. Significant consequences of midventricular obstruction and an aneurysmal apical chamber include ventricular arrhythmias, thromboembolism, predisposition to end stage disease and sudden cardiac death [1,3,5].

## Case Report

**Figure 1:**
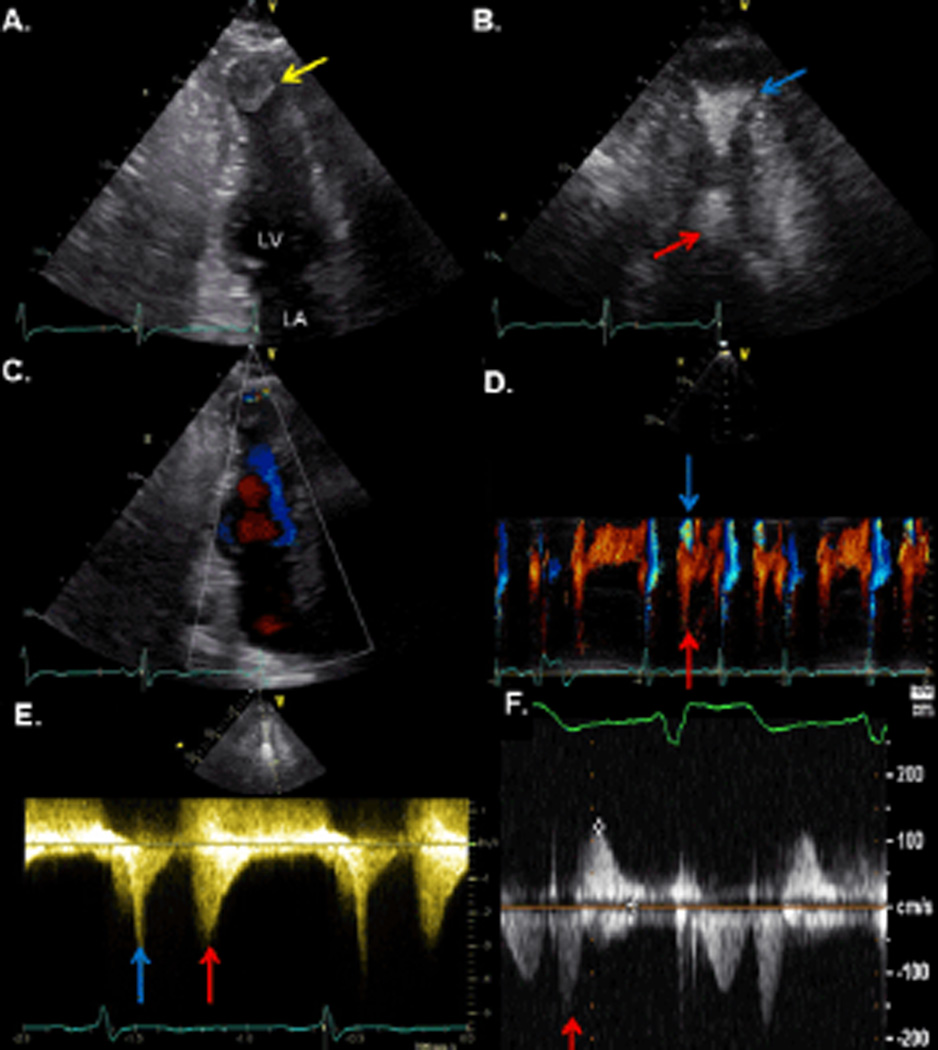
Two-dimensional transthoracic echocardiography and Doppler. A. The two-chamber view in mid diastole demonstrates a large mass at the left ventricular apex (arrow). B. Contrast-enhanced two chamber view in systole shows apposition of mid anterior and inferior segments resulting in an hourglass configuration of the left ventricle with separate apical (blue arrow) and basal chambers (red arrow). C. Color Doppler in early diastole reveals paradoxic flow (blue) from the apex toward the base due to blood previously trapped in apical chamber during systole. Red flow represents simultaneous, passive mitral inflow from the base toward the apex. D. Color M-mode confirms paradoxic flow (blue arrow) in early diastole and concurrent mitral inflow (red arrow). E. Contrast-enhanced continuous wave Doppler revealed a peak systolic velocity of 3.2 m/s (blue arrow) corresponding to a gradient of 41 mm Hg. Color Doppler (not shown) showed aliasing systolic flow with apposition of the midventricular segments, confirming the midcavitary level of obstruction. Continuous wave Doppler also demonstrated the paradoxic, early diastolic flow with a peak velocity of 2.7 m/s (red arrow) corresponding to a gradient of 29 mm Hg. F. Continuous wave Doppler performed postoperatively revealed no significant intracavitary obstruction. An insignificant jet of early diastolic flow (red arrow) persisted however. LA: Left atrium; LV: Left ventricle

A 70-year-old woman with hypertension, dyslipidemia and morbid obesity (43 kg/m^2^) status-post gastric bypass surgery over ten years prior to presentation was admitted for evaluation of an upper gastrointestinal bleed. Postoperatively following gastric bypass surgery, the patient became hemodynamically unstable and developed acute kidney injury. Although her renal function improved, the underlying etiology of her postoperative complications remained unknown. An abnormal electrocardiogram noted several years later suggested a prior myocardial infarction, retrospectively hypothesized incurred perioperatively. The abnormal electrocardiogram prompted invasive coronary angiography which showed no evidence of obstructive coronary artery disease. During the current admission for upper gastrointestinal bleeding, an abdominal CT scan with intravenous contrast serendipitously revealed a filling defect in the left ventricular apex. Transthoracic echocardiography confirmed the presence of a large, heterogeneous apical mass and additionally showed midventricular hypertrophy measuring 20 mm in the interventricular septum, resulting in systolic apposition of the midventricular segments (Figure 1A–B).

Contrast-enhanced continuous wave Doppler revealed paradoxic diastolic flow from the apex toward the base as well as a systolic midcavity gradient of 41 mmHg (Figure 1C–E). Cardiovascular MRI (CMR) was recommended for further evaluation of left ventricular structure, function and tissue characterization.

**Figure 2:**
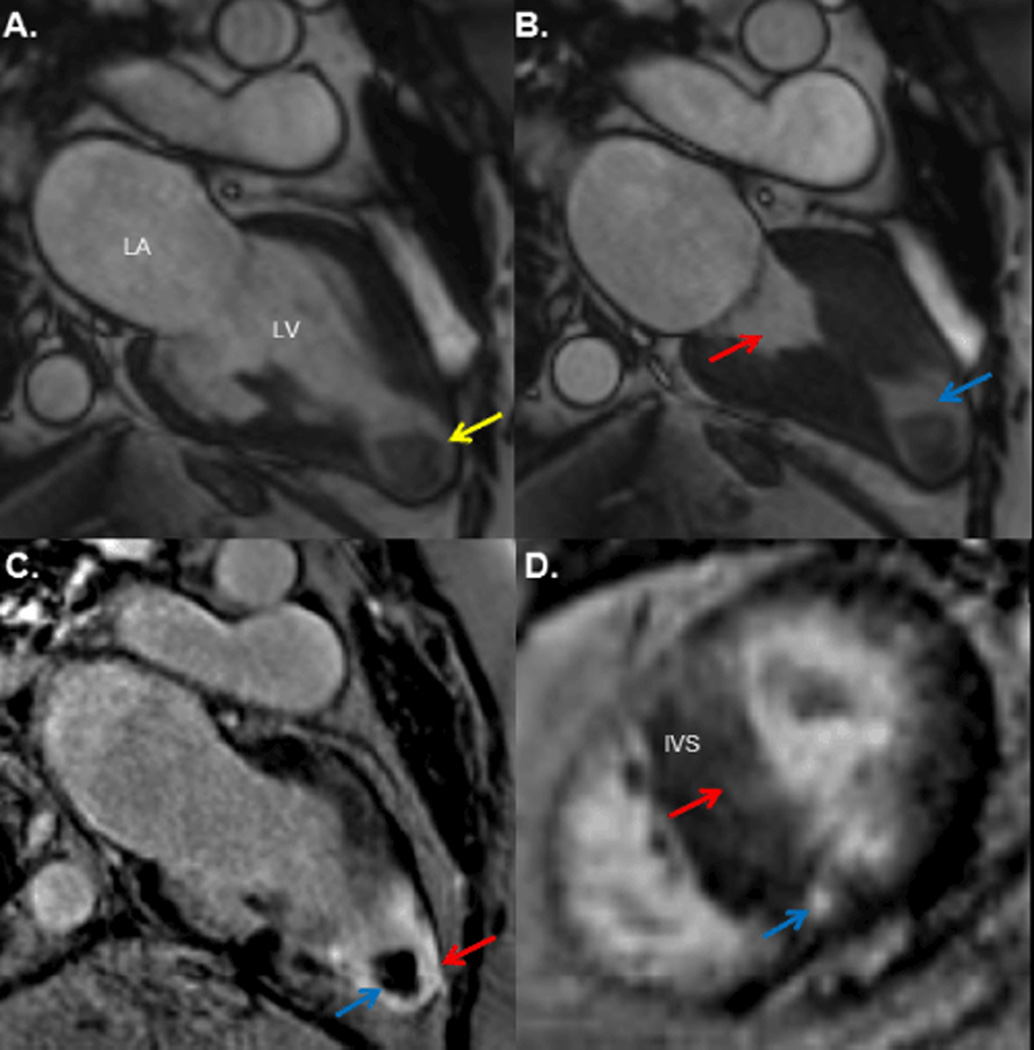
Cardiovascular magnetic resonance imaging. A. Steady-state free precession image of the two-chamber view at end diastole demonstrating a spade-shaped left ventricular cavity with mid ventricular hypertrophy and aneurysmal apex containing a mass (arrow) measuring 24 × 20 mm. B. The corresponding view at end systole demonstrates midventricular cavity obliteration resulting in separate apical (blue arrow) and ventricular chambers (red arrow). C. Late gadolinium enhancement imaging shows transmural fibrosis (bright) of the myocardium (red arrow). The periphery of the apical mass enhances (blue arrow) while the core remains hypointense (black), consistent with thrombus. D. Late gadolinium enhancement imaging of a short-axis mid ventricular view also demonstrates inferoseptal right ventricular insertion fibrosis (blue arrow) and patchy fibrosis of the interventricular septum (red arrow), consistent with hypertrophic cardiomyopathy. IVS: Interventricular septum; LA: Left atrium; LV: Left ventricle

CMR revealed a mildly dilated left ventricle (end diastolic volume index 99 mL/m^2^) with midcavity hypertrophy (maximal wall thickness 20 mm in the interventricular septum), a measured ejection fraction of 65% and an aneurysmal apex (Figure 2A).

Apposition of the midventricular segments in systole effectively resulted in separate apical and basal chambers (Figure 2B). The aneurysmal apical segments contained a large (24 × 20 mm) mass with peripheral enhancement and a hypointense core on late gadolinium enhancement imaging consistent with thrombus (Figure 2C). Late gadolinium enhancement also revealed a transmural myocardial infarction of the apical segments (Figure 2C), diffuse and patchy enhancement of the mid interventricular septum as well as focal enhancement of the mid inferoseptal right ventricular insertion point (Figure 2D). Native T1 mapping revealed an increased T1 relaxation time (1069 ms) in the mid septum compared to a lateral wall measurement (952 ms), consistent with increased relative fibrosis and the observed late gadolinium enhancement pattern. The extracellular volume fraction, measured in a region of interest localized to the interventricular septum on a mid-myocardial short-axis view, was found to be 44.6 ± 3.2% (mean ± standard deviation) (normal myocardium for our laboratory 25.4 ± 2.5%) [6], consistent with increased extracellular volume secondary to fibrosis.

**Figure 3:**
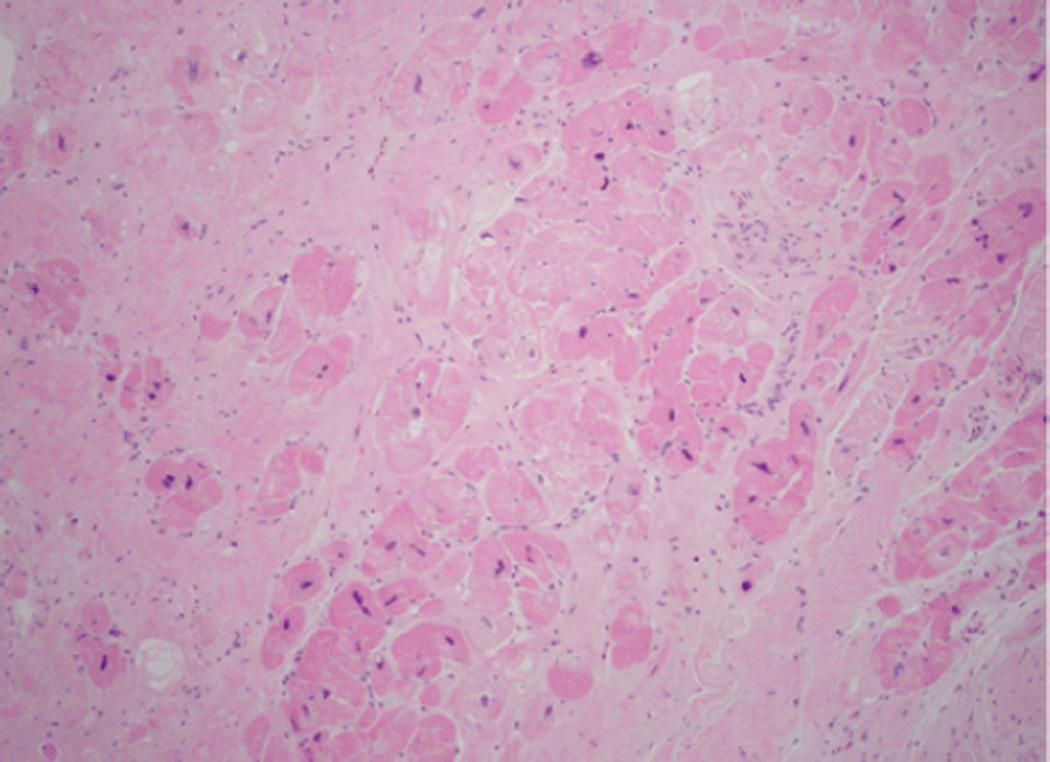
Histopathological evaluation of excised myocardium with hematoxylin and eosin staining revealed hypertrophied myocytes with hyperchromatic nuclei and fibrosis.

Due to the risk of embolization and the relative contraindication to anticoagulation given the recent gastrointestinal bleed, the patient underwent surgical thrombectomy as well as septal myectomy, aneurysm ligation and coronary artery bypass grafting to the right coronary artery. Preoperative invasive coronary angiography showed moderate right coronary artery disease, however no evidence of obstructive atherosclerosis to account for the apical infarction. Gross anatomic evaluation in the operating room confirmed the preoperative, noninvasive findings. The patient was found to have an apical thrombus with infarcted adjacent myocardium, midventricular hypertrophy and a large anterolateral papillary muscle, all consistent with hypertrophic cardiomyopathy and consequences of midventricular obstruction. Histological analysis confirmed that the apical mass consisted of thrombus. Evaluation of the left ventricular myocardial tissue showed hypertrophied myocytes with hyperchromatic nuclei and surrounding fibrosis (Figure 3), again consistent with the diagnosis of hypertrophic cardiomyopathy.

A postoperative transthoracic echocardiogram showed no evidence of left ventricular thrombus. Continuous wave Doppler of mitral inflow demonstrated no intracavitary obstruction, however a small, early diastolic gradient persisted (Figure 1F). A subsequent transthoracic echocardiogram performed after discontinuation of inotropic support showed no evidence of systolic or diastolic gradients. The patient’s hospital course and outpatient follow-up revealed no further bleeding or thrombotic complications.

## Discussion

### Diagnosis

This case potentially violates two major stipulations in the definition of HCM, including normal left ventricular chamber size and the absence of secondary causes of left ventricular hypertrophy. According to the 2011 ACCF/AHA Guideline for the Diagnosis and Treatment of Hypertrophic Cardiomyopathy, the disease is defined by “unexplained left ventricular hypertrophy associated with nondilated ventricular chambers in the absence of another cardiac or systemic disease that itself would be capable of producing the magnitude of hypertrophy evident in a given patient…” [1]. The guidelines further state, that in older patients with left ventricular hypertrophy and a history of systemic hypertension, the likelihood of hypertrophic cardiomyopathy can be inferred by left ventricular wall thickness >25 mm. Therefore, the patient fails to meet guidelines for the diagnosis of HCM based on a dilated left ventricle, history of hypertension and a maximal wall thickness of <25 mm. Given the violation of these major stipulations in the definition of HCM, the integrated use of all diagnostically useful information obtained from multimodality imaging is of paramount importance. The differential diagnosis should therefore also include hypertensive heart disease, myocardial infarction with spontaneous lysis of thrombus and other cardiomyopathies. However, when considering the clinical context associated with the patient’s hemodynamic instability at the time of bariatric surgery, the absence of obstructive coronary artery disease, the paradoxic diastolic flow pattern noted on Doppler echocardiography, the morphology and scar pattern of the left ventricle on CMR, the most likely unifying diagnosis remains HCM with midventricular obstruction and aneurysmal apical chamber.

### Pathogenesis and pathophysiology

We postulate that at the time of bariatric surgery under generalized anesthesia, the patient experienced relative hypotension, resulting in decreased afterload, exacerbation of the midventricular obstruction and a corresponding decrease in cardiac output. The increased midventricular gradient caused increased myocardial wall tension (according to Laplace’s law), oxygen supply-demand mismatch, ischemia and ultimately infarction and fibrosis. Patients with HCM also have abnormally thickened intramural arteries with decreased luminal size [7]. Coupled with significant midventricular obstruction, these HCM patients are particularly predisposed to ischemia, infarction and fibrosis. Over time, multiple small infarctions may lead to a confluent scar similar to that resulting from a distal left anterior descending coronary artery myocardial infarction. An infarcted apex may lead to ventricular remodeling, dilation, aneurysm and predisposition to thrombus formation [8], as exhibited by the patient described.

The increasingly utilized CMR technique of native T1 mapping characterizes myocardium based on quantitative T1 relaxation times, altered by the presence of fibrosis, without the need for intravenous gadolinium contrast agent. Increased T1 relaxation times in hypertrophic cardiomyopathy correlate with abnormalities in left ventricular systolic function even in the absence of late gadolinium enhancement [9]. In the patient described, the elevated T1 relaxation time noted in the interventricular septum correlates with the patchy late gadolinium enhancement pattern observed. Similarly, the technique of extracellular volume fraction mapping quantifies the fraction of extracellular volume within a myocardial region of interest [6]. Increased fibrotic tissue also explains the elevated extracellular volume fraction observed within the interventricular septum.

Patients with HCM and midventricular obstruction may exhibit a characteristic echocardiographic Doppler pattern of blood flow in the left ventricle [10]. Due to the midventricular obstruction, there are effectively two separate chambers within the left ventricle at end systole. The blood that pools in the apical chamber may paradoxically flow toward the base in early diastole, resulting in a diastolic flow signal that is not ordinarily seen in normal hearts. The Doppler profile observed here (Figure 1C–F) indicates this paradoxical early diastolic flow from the apex toward the base. Interestingly, patients with evidence of paradoxical jet flow more often develop systemic embolism compared to those with cavity obliteration alone. Although not observed in this case, we parenthetically note that a coronary artery-LV fistula, a rare association with apical HCM, may also result in apical diastolic flow [11].

### Prognosis

This morphological variant of HCM deserves clinical recognition. In a Japanese study [2] of 490 consecutive patients with HCM, the presence of midventricular obstruction was found to be an independent predictor of HCM-related death (hazard ratio 2.23, p=0.016). Apical aneurysm was identified in 28.3% of patients with midventricular obstruction and predicted HCM-related death (hazard ratio 3.47, p=0.008). Interestingly, midventricular obstruction without an apical aneurysm was not an independent determinant of HCM-related death overall (adjusted hazard ration 1.72, p=0.213).

Maron et al. [12] have questioned the mechanism of aneurysm formation in patients with HCM and midcavitary obstruction. Of 1299 patients with HCM, 28 (2%) had apical aneurysms and only 10 of these 28 patients demonstrated intracavitary gradients (25–150 mm Hg). Therefore, increased wall tension secondary to an intracavitary gradient may not be the only mechanism to explain left ventricular aneurysm formation. Over 4.1 ± 3.7 years of follow-up, 12 patients (43%) with left ventricular apical aneurysms experienced adverse disease-related complications, an annual event rate of 10.5%, which is significantly higher than the 1.3% reported in the general HCM population [13]. Consequences of aneurysm formation included ventricular arrhythmias, thromboembolism, predisposition to end stage disease and sudden death. Patients with larger apical aneurysms were more likely to experience disease-related complications. The 2011 ACCF/AHA Guideline for the Diagnosis and Treatment of Hypertrophic Cardiomyopathy suggests that the presence of an apical aneurysm may warrant consideration in sudden cardiac death risk assessment [1].

## Conclusions

Hypertrophic cardiomyopathy with midventricular hypertrophy and obstruction is a relatively uncommon variant of HCM that may lead to an infarcted apex and aneurysm formation. Mid ventricular obstruction with apical aneurysm formation predisposes to thrombus formation and portends a poor prognosis. Repercussions of aneurysm formation included ventricular arrhythmias, thromboembolism, predisposition to end stage disease and death. Physicians should be aware of this variant and consider these patients to be at higher risk when making management decisions.
